# Dataset on body weight and length of rainbow trout, *Oncorhynchus mykiss*, fed with dihydroquercetin, arabinogalactan or a mixture of both in an aquaria experiment

**DOI:** 10.1016/j.dib.2020.106184

**Published:** 2020-08-16

**Authors:** Nadezhda Kantserova, Ekaterina Borvinskaya, Liudmila Lysenko, Irina Sukhovskaya, Maria Churova, Ekaterina Tushina

**Affiliations:** Institute of Biology, Karelian Research Centre of Russian Academy of Sciences, Petrozavodsk 185910, Russian Federation

**Keywords:** Rainbow trout, Growth, Aquaria experiment, Dietary supplement, Dihydroquercetin, Arabinogalactan

## Abstract

The use of natural dietary supplements in aquaculture has received a great deal of attention in recent years. This article provides data describing body weight and length of rainbow trout juveniles fed with natural dietary supplements dihydroquercetin, arabinogalactan or a mixture of both in an aquaria experiment. Before feeding trial, rainbow trout were tagged to identify individuals. Fish grown in tanks were fed one of four diets in duplicate: a basal diet without any supplements (control diet) or a basal diet supplemented with dihydroquercetin (experimental diet 1), arabinogalactan (experimental diet 2) or a mixture of both (experimental diet 3). Our dataset could be used to evaluate the effect of dihydroquercetin, arabinogalactan or a mixture of both on the growth performance of cultivated rainbow trout.

**Specifications Table**

**Table:** 

**Subject**	Aquatic Science
**Specific subject area**	Aquaculture nutrition, growth performance of cultivated fish
**Type of data**	Table and Figure
**How data were acquired**	Rainbow trout body weight and length were measured with a digital balance (accuracy: 0.01 g; model SPX2202, OHAUS Corporation, USA) and a 300 mm liquid crystal display (LCD) digital Vernier caliper (accuracy: 0.01 cm; CHIZ, Russia), respectively. Data were analyzed with the lmer function from the lme4 package for R.
**Data format**	Raw Analyzed
**Parameters for data collection**	Body weight and length measurement of tagged rainbow trout was conducted before and during the feeding trial. Fish grown in tanks were fed one of four diets in duplicate groups: a basal diet without any supplements (control diet) or a basal diet supplemented with dihydroquercetin (experimental diet 1), arabinogalactan (experimental diet 2) or a mixture of both (experimental diet 3).
**Description of data collection**	124 rainbow trout juveniles (age: 6 months) were included in aquaria experiment. Control group included 32 fish in two tanks (no. 1, n_1_=16; no. 8, n_2_=16), experimental group 1 included 30 fish in two tanks (no. 5, n_1_=14; no. 3, n_2_=16), experimental group 2 included 32 fish in two tanks (no. 4, n_1_=16; no. 7, n_2_=16), experimental group 3 included 34 fish in two tanks (no. 2, n_1_=17; no. 6, n_2_=18) (Fig. 1). Before feeding trial, rainbow trout were PIT tagged to identify individuals. Fish tagging and body weight and length measurement followed by anesthesia using a clove oil bath. Rainbow trout body weight and length measurements were conducted with an accuracy of 0.01 g and 0.01 cm, respectively.
**Data source location**	Laboratory for Environmental Biochemistry, Institute of Biology of Karelian Research Center of the Russian Academy of Sciences, Petrozavodsk, Russia. Rainbow trout, *O. mykiss*, juveniles were obtained from the commercial trout farm, Ladmozero Lake, Republic of Karelia, Russia.
**Data accessibility**	With the article

**Value of the Data**

• The presented data give details on the effect of dietary dihydroquercetin (25 and 1000 mg kg⁻¹ of diet), arabinogalactan (50 and 2000 mg kg⁻¹ of diet) or their combination (25+50 and 1000+2000 mg kg⁻¹ of diet) on the weight and length of rainbow trout in an aquaria experiment.
• These data could be taken into account by fish farmers in the estimation of the effect of natural dietary supplements such as dihydroquercetin and arabinogalactan on rainbow trout growth. These data will be helpful for researchers involved in aquaculture nutrition assessments and related research.
• These data support the development of further studies aimed to reveal concentrations of dihydroquercetin and arabinogalactan that may affect the growth performance of farmed rainbow trout.
• These data confirm the current knowledge on the growth pattern of rainbow trout juveniles.

## 1. Data description

Fig. 1

**Fig. 1:**
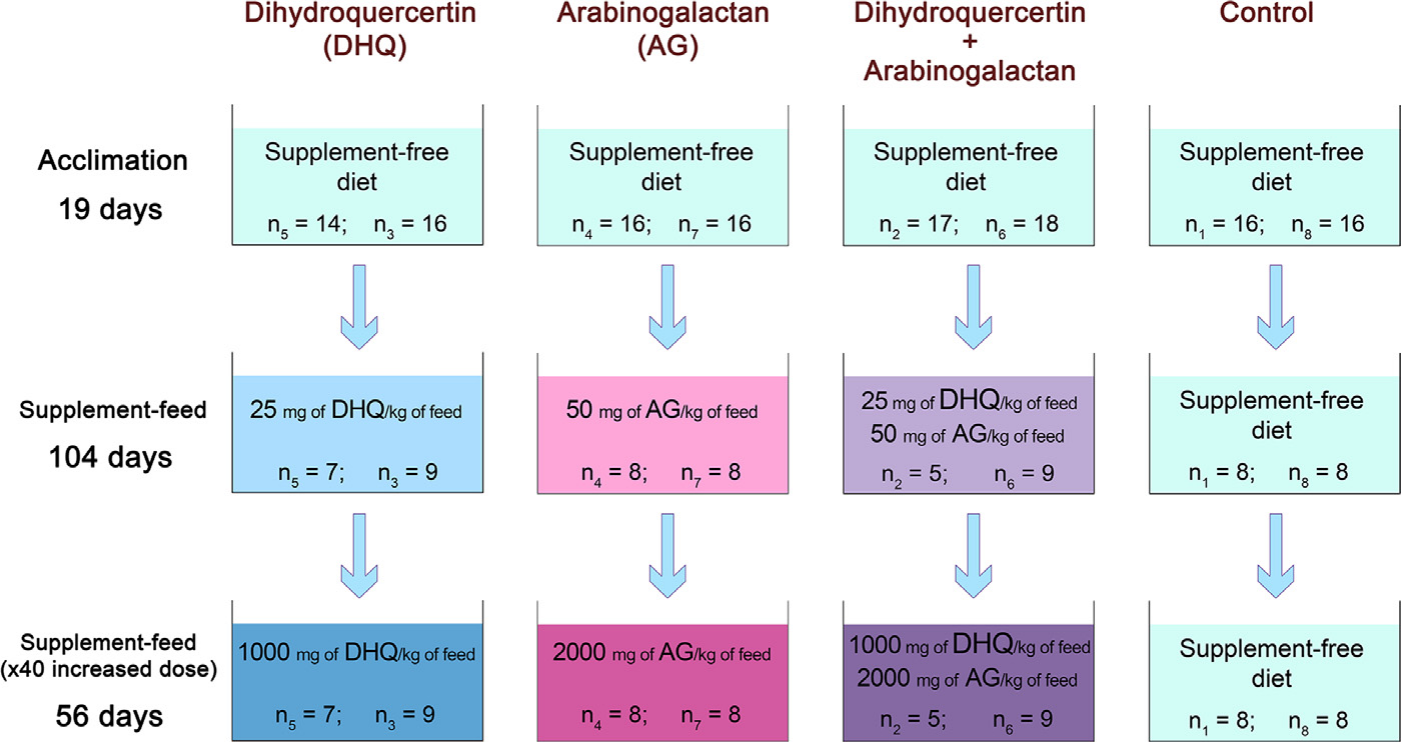
The design of the aquaria experiment. The total number of tanks was 8 (2 tanks in each group); n_х_ denotes the number of fish, where _x_ is the no. of the tank.

**Fig. 2:**
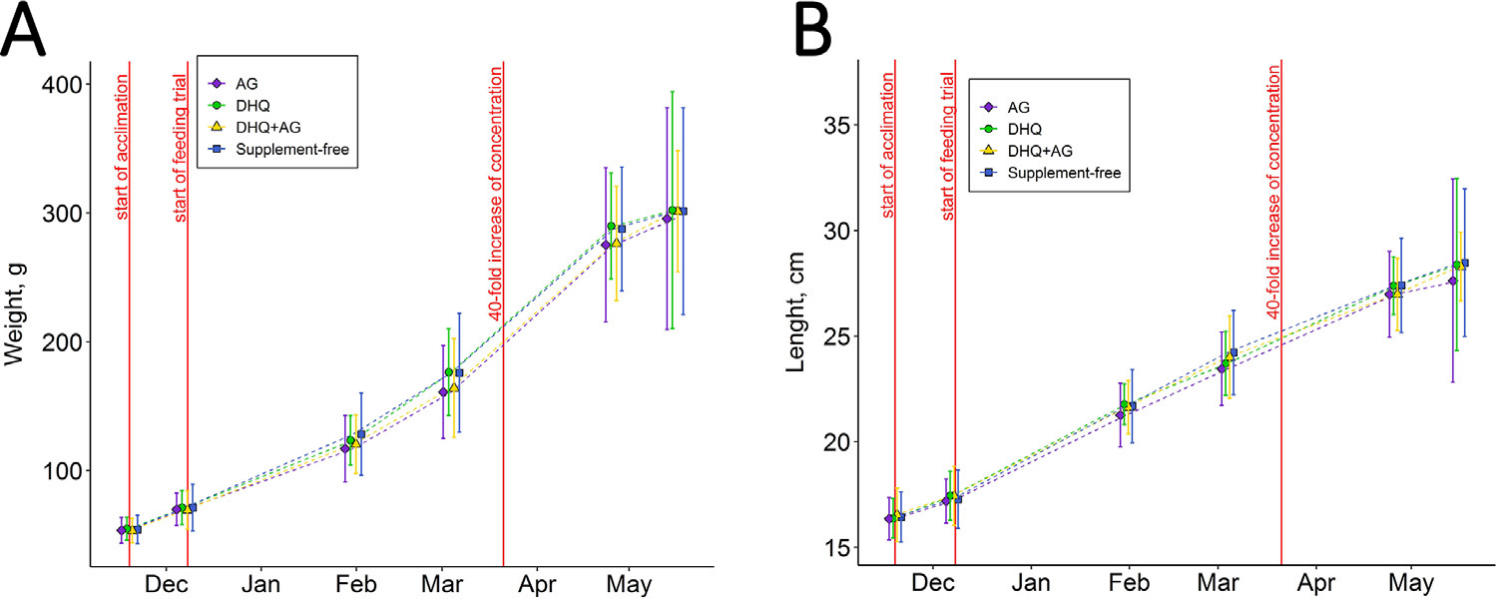
Weight (A) and length (B) of rainbow trout fed with a diet without any supplements (control diet) or a diet supplemented with dihydroquercetin (DHQ), arabinogalactan (AG) or a mixture of both (DHQ+AG) (F-test, *p* ≤ 0.05).

The dataset presented in this article as a supplementary file (.xls) provides raw data on the growth of rainbow trout individuals fed with dihydroquercetin, arabinogalactan or a mixture of both in an aquaria experiment. It has 7 columns: tag number, measurement date, weight (g), length (cm), group (control, dihydroquercetin-fed, arabinogalactan-fed, or a mixture of dihydroquercetin and arabinogalactan-fed), the concentration of supplements (mg kg⁻¹ of feed), and tank number. We applied multilevel linear modeling for repeated measures data, which revealed no difference in growth parameters (body weight and length) between the control and experimental groups. Data extracted from this experiment are summarized in Fig. 2. No significant differences in weight (Fig. 2A) or length (Fig. 2B) between studied groups were found.

## 2 Experimental design, materials, and methods

### 2.1 Experimental system and fish rearing

All animal handling procedures were approved by the Ethics and Animal Care Committee of the Institute of Biology, Karelian Research Center of the Russian Academy of Sciences, following EU-established norms and procedures.

On November 1, 2018 rainbow trout, *O. mykiss*, juveniles (age: 6 months) obtained from a commercial trout farm (Ladmozero Lake, Republic of Karelia, Russia) were transported to the Laboratory for Environmental Biochemistry at the Institute of Biology, Karelian Research Center of the Russian Academy of Sciences (Petrozavodsk, Russia). Fish were randomly stocked into 8 glass tanks (250 - 270 L capacity) with 14–17 fish per tank. Tanks were continuously supplied with aerated water with the flow rate set at 0.16 L min⁻¹, water temperature 12 ± 1 °C, dissolved oxygen 7.5–8.5 ppm, total ammonia nitrogen < 0.1 mg *L* ^−^ ^1^, nitrite nitrogen < 0.1 mg *L* ^−^ ^1^ and nitrate nitrogen < 10.0 mg *L* ^−^ ^1^, under natural photoperiod. On November 19, 2018 fish were PIT tagged intraperitoneally to identify individuals. Fish were anesthetized using a clove oil bath (25–30 mg L⁻¹) before tagging as well as before all subsequent body weight and length measurements. On November 19, 2018 stocking density was 3.24, 3.25, 3.39, 3.24, 3.12, 3.60, 3.29, 3.37 kg *m* ^−^ ^3^ for tanks from 1 to 8, respectively. Before the start of the feeding trial, all fish were given a basal diet, EFICO Alpha 717R (BioMar, Denmark), containing 22–25% lipid, 40–43% protein, 20–23% carbohydrate, 2.8–5.8% fiber, 0.9% total P, 4–7% ash and 22–25 MJ kg⁻¹ total energy. Tagged fish were acclimatized to the experimental conditions for 19 days prior to the start of the feeding trial.

### 2.2 Diet and feeding protocol

Dihydroquercetin, a bioflavonoid, («Lavitol-dihydroquercetin», certificate no. 396–08.17) and arabinogalactan, a polysaccharide, («Lavitol-arabinogalactan», certificate no. 452–08.17) were purchased from Ametis (Russia). Distilled water was used to dissolve supplements; then the solution was heated up to 45 °C and stirred continuously for 1 h. Visual control of the dissolution process made it possible to make sure that there was no sediment. The solution with supplements was sprayed onto feed pellets from a spray gun directly on the day of feeding. Then the feed pellets were dried at room temperature for an hour. In the control group, distilled water alone was added to the feed.

The feeding trial was started on December 8, 2018. Fish were fed one of four diets in duplicate tanks: a basal diet without any supplements (control diet) or a basal diet supplemented with dihydroquercetin (25 mg kg⁻¹ of diet; experimental group 1), arabinogalactan (50 mg kg⁻¹ of diet; experimental group 2) or a mixture (25+50 mg kg⁻¹ of diet, respectively; experimental group 3). The supplement concentrations listed above were recommended by the manufacturer (Ametis, Russia) for fish farming. Fish were fed these diets until March 20, 2019. From March 21, 2019 until May 15, 2019, fish were fed diets with a 40-fold increase of the initial supplement concentration: a basal diet without any supplements (control diet), or a basal diet supplemented with dihydroquercetin (1000 mg kg⁻¹ of diet; experimental group 1), arabinogalactan (2000 mg kg⁻¹ of diet; experimental group 2) or a mixture of both (1000+2000 mg kg⁻¹ of diet, respectively; experimental group 3). High concentrations of dihydroquercetin (1000, 5000 and 10,000 mg kg⁻¹ of diet) have been used previously in the experiment with gilthead seabream with no toxicity for fish [1]. Fish were fed once a day; the feeding level based on percent of tank biomass was equal for experimental and control groups. The food was eaten completely. Pellet size of the diet was equal for all groups. From November 1, 2018 until March 1, 2019 pellet size of the diet was 3 mm, and from March 1, 2019 it was 4 mm.

### 2.3 Growth parameters

Rainbow trout body weight and length measurements were taken on November 19, 2018; December 7, 2018; January 31, 2019; March 4, 2019; April 26, 2019; and May 16, 2019 with a digital balance (accuracy: 0.01 g; model SPX2202, OHAUS Corporation, USA) and a 300 mm liquid crystal display (LCD) digital Vernier caliper (accuracy: 0.01 cm; CHIZ, Russia), respectively. Up to 10 fish from each tank were sacrificed on December 7, 2018 for further biochemical analysis (Kantserova et al., unpublished), so for these fish, growth parameter data is only available for two dates (November 19, 2018 and December 7, 2018). On December 8, 2018 stocking density was 2.16, 1.52, 2.34, 2.00, 1.82, 2.12, 2.21, 2.15 kg *m* ^−^ ^3^ for tanks from 1 to 8, respectively. During the study, some healthy fish were lost as a result of accidents (e.g., a fish jumped out of an aquarium). These accidents involved fish with tags nos. 330 (01.01.2019), 486 (11.01.2019) and 677 (18.01.2019) from tank no. 2, fish with tag no. 913 (08.01.2019) from tank no. 3, and fish with tag no. 924 (06.12.2019) from tank no. 5. The remaining fish having two measures (19.11.2018, 07.12.2019) were used for biochemical analysis.

### 2.4 Experimental data analysis

The analysis of repeated measures data was performed utilizing a multilevel linear model (MLM). The data were analyzed with the lmer function from the lme4 package [2] for R [3]. The differences between the experimental groups were evaluated using F- and t-tests for the objects returned by a mixed model function with a p value cutoff of 0.05 for statistical significance.

## Declaration of Competing Interest

The authors declare that they have no known competing financial interests or personal relationships which have, or could be perceived to have, influenced the work reported in this article.

## Appendix Supplementary materials

Image, application 1

Image, application 2
